# Supplementary material for: A local structural basis to distinguish amorphous ices
Source: Nat Commun. 2026 Sep 12;17:10754. doi: 10.1038/s41467-026-77279-5 (PMC13661517; doi:10.1038/s41467-026-77279-5)
Supplement: Supplementary file 1 [file 41467_2026_77279_MOESM1_ESM.pdf]

Supporting Information for "A Local Structural Basis to Distinguish Amorphous Ices"

Quinn M. Gallagher,[1,*] Ryan J. Szukalo*,[2,†] Nicolas Giovambattista,[3,4] Pablo G. Debenedetti,[1] and Michael A. Webb[1,‡]

[1]*Department of Chemical and Biological Engineering, Princeton University, Princeton, NJ 08540, United States*
[2]*Department of Chemistry, Princeton University, Princeton, NJ 08540, United States*
[3]*Department of Physics, Brooklyn College of the City University of New York, Brooklyn, New York 11210, United States*
[4]*Ph.D. Programs in Physics and Chemistry, The Graduate Center of the City University of New York, New York, New York 10016, United States*

CONTENTS

S1. Methods 2
A. Structural Descriptors 2
1. Atom-Centered Symmetry Functions 2
2. Bond-Orientational Order Parameters 3
B. Automatic Feature Selection 4
C. Probabilistic Model 5
D. Training Data and Simulation Details 6

S2. Benchmarking Model Performance for DP_SCAN Environments 6

S3. Out-of-distribution Predictions for DP_SCAN and DP_MBpol Environments 8

S4. Out-of-distribution Prediction for Alternative Phases of Ice 9

S5. Sensitivity of Structural Pathways to Selected Feature Spaces 10

S6. Differences between DP_SCAN- and DP_MBpol-generated HDA Environments 12

References 13

* These authors contributed equally to this work.
† rszukalo@princeton.edu
‡ mawebb@princeton.edu

S1. METHODS

Figure 1 in the main text illustrates our framework that combines interpretable structural descriptors with probabilistic classification to identify local collective variables that distinguish amorphous phases. First, each molecule's local environment is represented using atom-centered symmetry functions (ACSFs) and bond-orientational order (BOO) parameters, which together provide a comprehensive, symmetry-invariant characterization of local structure (Step 1). Second, a two-stage feature selection procedure uses mutual information (MI) to rank descriptors by discriminative power and removes redundant descriptors through correlation analysis (Step 2). Third, class-conditional probability distributions of the selected descriptors are then modeled, enabling transparent probabilistic classification (Step 3). Finally, the joint probability for each class is used for molecule-by-molecule state assignment and explicit detection of out-of-distribution configurations, i.e., molecular environments that cannot be classified as LDA-like or HDA-like (Step 4). This approach automatically identifies which structural descriptors best distinguish the amorphous ices without requiring *a priori* knowledge of the relevant order parameters, providing both high classification accuracy and physical interpretability. The following sections detail each step of this procedure.

A. Structural Descriptors

We consider two complementary, interpretable descriptor families that together provide a comprehensive characterization of microscopic environments. ACSFs encode the local environment through radially and angularly resolved correlations of interatomic distances [1]. BOO parameters [2] provide a complementary description that quantifies the orientational symmetry of the surrounding molecular arrangement and have been widely used to characterize amorphous and crystalline ices [3, 4]. Together, these descriptors form a symmetry-invariant and physically grounded representation of each molecule's local environment (Fig. 1, Step 1).

1. Atom-Centered Symmetry Functions

ACSFs are computed using several distinct functional forms that capture complementary aspects of local atomic environments through radially and angularly resolved correlations. All ACSF functions are centered on oxygen atoms (central oxygen atom i), with contributions included from both oxygen and hydrogen neighbors to ensure sensitivity to variations in hydrogen-bond motifs and proton arrangements not visible from oxygen positions alone. Throughout, we denote $\mathbf{r}_{ij}$ as the displacement vector from central atom i to neighboring atom j and $R_{ij} = \|\mathbf{r}_{ij}\|$ as the corresponding scalar distance. All ACSF functions employ a smooth cutoff function $f_\mathrm{c}(R_{ij})$:

$$f_\mathrm{c}(R_{ij}) = \begin{cases} 0.5\left[\cos\left(\frac{\pi R_{ij}}{R_\mathrm{c}}\right) + 1\right] & \text{if } R_{ij} \leq R_\mathrm{c} \\ 0 & \text{if } R_{ij} > R_\mathrm{c}, \end{cases} \tag{1}$$

This cutoff function smoothly decays from 1 at $R_{ij} = 0$ to 0 at $R_{ij} = R_\mathrm{c}$, with both the function and its first derivative vanishing continuously at the cutoff radius. We define the local environment around a central oxygen i by the 16 nearest neighboring water molecules. Let $\mathcal{N}_\mathrm{mol}(i)$ denote this set of 16 neighbor molecules. We then define the corresponding sets of neighboring atoms as $\mathcal{N}_\mathrm{O}(i)$ and $\mathcal{N}_\mathrm{H}(i)$, the set of the 16 neighboring oxygen atoms and 32 neighboring hydrogen atoms belonging to the molecules in $\mathcal{N}_\mathrm{mol}(i)$. Unless otherwise stated, we set $R_\mathrm{c} \approx 5.0\,\text{Å}$ and always ensure that all atoms in these neighbor sets satisfy $R_{ij} \leq R_\mathrm{c}$ (*i.e.*, the neighbor list is defined by neighbor count).

Three types of radial symmetry functions provide complementary representations of the local density. The simplest radial function, G^1, provides a weighted count of neighboring atoms:

$$G^1 = \sum_{j \in \mathcal{N}_\gamma(i)} f_\mathrm{c}(R_{ij}), \tag{2}$$

where $\gamma \in \{\mathrm{O}, \mathrm{H}\}$ specifies the neighbor species set. In particular, we compute separate G^1 functions for oxygen and hydrogen neighbors, denoted G^1_O and G^1_H.

The next function, G^2, focuses on the local density at a particular distance from the central atom,

$$G^2(\eta, R_\mathrm{s}) = \sum_{j\in\mathcal{N}_\gamma(i)} \exp\left[-\eta(R_{ij} - R_\mathrm{s})^2\right] f_\mathrm{c}(R_{ij}), \tag{3}$$

where η controls the width of the Gaussian and R_s shifts its center. By using multiple G^2 functions with different η and R_s values, these functions can construct a radially resolved representation of the local density.

The third function, G^3, utilizes cosine damping:

$$G^3(\kappa) = \sum_{j\in\mathcal{N}_\gamma(i)} \cos(\kappa R_{ij}) f_\mathrm{c}(R_{ij}), \tag{4}$$

where κ adjusts the period length. Using multiple G^3 functions with different κ values provides a Fourier-like decomposition of the radial distribution.

Two types of angular functions capture information about three-body configurations and local orientational order. This is achieved by considering angles centered at atom i formed by neighbor atoms j and k,

$$\theta_{jik} = \arccos\left(\frac{\mathbf{r}_{ij}\cdot\mathbf{r}_{ik}}{R_{ij}R_{ik}}\right). \tag{5}$$

The two functions take the form (summing over unordered neighbor pairs $j < k$):

$$G^4(\eta,\zeta,\lambda) = 2^{1-\zeta} \sum_{j\in\mathcal{N}_\alpha(i)} \sum_{\substack{k\in\mathcal{N}_\beta(i)\\ k>j}} (1+\lambda\cos\theta_{jik})^\zeta \times \exp[-\eta(R_{ij}^2 + R_{ik}^2 + R_{jk}^2)] f_\mathrm{c}(R_{ij}) f_\mathrm{c}(R_{ik}) f_\mathrm{c}(R_{jk}), \tag{6}$$

and

$$G^5(\eta,\zeta,\lambda) = 2^{1-\zeta} \sum_{j\in\mathcal{N}_\alpha(i)} \sum_{\substack{k\in\mathcal{N}_\beta(i)\\ k>j}} (1+\lambda\cos\theta_{jik})^\zeta \times \exp[-\eta(R_{ij}^2 + R_{ik}^2)] f_\mathrm{c}(R_{ij}) f_\mathrm{c}(R_{ik}). \tag{7}$$

Here, $\alpha, \beta \in \{\mathrm{O}, \mathrm{H}\}$ specify the neighbor species sets used for j and k. The parameter ζ controls the angular resolution, with larger values emphasizing specific angular arrangements, while $\lambda \in \{-1, 1\}$ shifts the sensitivity to linear ($\lambda = -1$) versus bent ($\lambda = 1$) configurations. The parameter η controls the radial extent over which angular correlations are evaluated. The key distinction between G^4 and G^5 lies in their radial dependencies. G^4 includes the neighbor–neighbor distance R_{jk}, restricting contributions to compact triplets where all three interatomic distances are small, while G^5 omits this constraint and thus captures angular correlations over a wider range of geometries. These angular functions are computed over all possible species combinations of the three atoms involved, with parameter sets chosen to capture angular correlations at different radial extents.

2. Bond-Orientational Order Parameters

BOO parameters provide a complementary description of local structure by quantifying orientational symmetry through spherical harmonics [2]. Unlike ACSFs which are explicitly constructed as sums over neighbors, BOO parameters characterize the angular distribution of the local environment in a rotationally invariant manner. For each oxygen atom i, we use the same oxygen neighbor list as above and define $N_\mathrm{b} = |\mathcal{N}_\mathrm{O}(i)| = 16$ neighboring oxygen atoms. The complex-valued order parameter q_{lm} is computed as:

$$q_{lm}(i) = \frac{1}{N_\mathrm{b}} \sum_{j\in\mathcal{N}_\mathrm{O}(i)} Y_{lm}(\theta_{ij}, \phi_{ij}), \tag{8}$$

where Y_{lm} are spherical harmonics of degree l and order m and (θ_{ij}, ϕ_{ij}) are the polar and azimuthal angles of the vector $\mathbf{r}_{ij}$ connecting oxygen atoms i and j. The Steinhardt BOO parameter q_l is obtained by combining all m components for a given l:

$$q_l(i) = \sqrt{\frac{4\pi}{2l+1} \sum_{m=-l}^{l} |q_{lm}(i)|^2}. \tag{9}$$

Low values of l (e.g., $l = 3, 4$) are sensitive to tetrahedral and other low-symmetry arrangements, while higher l values capture more complex orientational motifs.

We also include third-order descriptors w_l, which provide additional sensitivity to the shape of the orientational distribution:

$$w_l(i) = \frac{\sum_{m_1+m_2+m_3=0} C_l^{m_1 m_2 m_3}\, q_{lm_1}(i)\, q_{lm_2}(i)\, q_{lm_3}(i)}{\left(\sum_{m=-l}^{l} |q_{lm}(i)|^2\right)^{3/2}}, \tag{10}$$

where $C_l^{m_1 m_2 m_3}$ are the Wigner 3-j coupling coefficients and the sum runs over all triplets (m_1, m_2, m_3) satisfying $m_1 + m_2 + m_3 = 0$. The w_l parameters are particularly useful for distinguishing structures with similar q_l values but different higher-order orientational correlations.

Additionally, we compute locally averaged variants that incorporate information from the neighborhood surrounding each molecule [5]. The locally averaged complex order parameter $\bar{q}_{lm}(i)$ is defined as:

$$\bar{q}_{lm}(i) = \frac{1}{N_{\mathrm{b}}+1} \left[q_{lm}(i) + \sum_{j \in \mathcal{N}_{\mathrm{O}}(i)} q_{lm}(j) \right]. \tag{11}$$

This averaging procedure includes contributions from both the central molecule i and its N_{b} oxygen neighbors. From $\bar{q}_{lm}(i)$, we compute the rotationally invariant

$$\bar{q}_l(i) = \sqrt{\frac{4\pi}{2l+1} \sum_{m=-l}^{l} |\bar{q}_{lm}(i)|^2}. \tag{12}$$

The locally averaged descriptors $\bar{q}_l$ provide enhanced discrimination between crystal structures compared to their unaveraged counterparts q_l, as the averaging reduces thermal fluctuations while preserving sensitivity to orientational order [5]. The corresponding locally averaged third-order descriptors $\bar{w}_l$ are computed using Eq. (10) but with all $q_{lm}(i)$ substituted by $\bar{q}_{lm}(i)$.

Together with the ACSF descriptors, this comprehensive set of structural descriptors provides a detailed, physically interpretable representation of each oxygen-centered local environment.

B. Automatic Feature Selection

We extract the most informative descriptors from the full set using an automated ranking procedure (Fig. 1, Step 2). We define local environments using the 16 nearest neighbors, consistent with prior work [3, 4, 6]. We assess the ability of each descriptor to discriminate between classes (LDA-like and HDA-like) using mutual information (MI) [7]. MI quantifies the reduction in uncertainty about the class label upon observing a descriptor value and thus provides a model-free measure of discriminative power. For a feature X and class label Y, where x and y denote particular values of X and Y respectively, the MI is defined as:

$$\mathrm{MI}(X;Y) = \sum_{x \in X} \sum_{y \in Y} p(x,y) \log \frac{p(x,y)}{p(x)p(y)}, \tag{13}$$

where $p(x, y)$ is the joint probability density of feature X and class label Y, and $p(x)$ and $p(y)$ are the corresponding distributions. We rank features by MI and retain those with MI values of at least 10% of the maximum observed MI, such that all features are expected to possess useful information.

To ensure that selected descriptors contribute distinct structural information, we use a greedy high-correlation filter. Proceeding in order of decreasing MI, each descriptor is retained only if its Pearson correlation (r) with all previously retained descriptors satisfies $|r| < 0.8$. The outcome is a set of informative and roughly uncorrelated descriptors, which we denote $\mathcal{D} = \{1, \ldots, M\}$. Because the procedure operates on arbitrary descriptor inputs, it enables data-driven identification of discriminative descriptors without requiring prior assumptions about which structural motifs are important. We retain $\mathcal{D}$ in its entirety for out-of-distribution detection (see Probabilistic Model, below), since each additional weakly correlated descriptor offers an independent opportunity to identify an anomalous environment and thus maximizes detection sensitivity. For classification, however, the number of descriptors retained from $\mathcal{D}$ is a user-specified parameter; here we select the $m = 5$ most informative descriptors, denoted $\mathcal{D}_{\rm cls} = \{1, \ldots, m\} \subset \mathcal{D}$, balancing classification accuracy with physical interpretability. The full set of ACSF and BOO descriptors considered as candidates spans all functional forms defined in Section S1 A, and the specific descriptors selected for LDA/HDA classification are shown in Figure 2E of the main text.

C. Probabilistic Model

The second component of our framework is a probabilistic classifier that assigns a label to each atomic environment as a calibrated likelihood of belonging to each structural class (Fig. 1, Step 3). Rather than learning a decision boundary, we model the probability distribution of each selected descriptor for each class.

To avoid index ambiguity, we use $a \in \{1, \ldots, m\}$ to index the selected m features in $\mathcal{D}_{\rm cls}$ used for classification and $n \in \{1, \ldots, N_y\}$ to index training samples within class y. For each feature a and class y, we estimate the corresponding one-dimensional probability density $p_{a,y}(x)$ of observing value x using Gaussian kernel density estimation (KDE). In k-fold cross-validation, the dataset is partitioned into k equal subsets. The model is trained on $k-1$ subsets and evaluated on the held–out subset, with this procedure repeated k times so that every sample serves as a validation point exactly once. Performance metrics are then averaged across all k folds, providing a robust estimate of generalization performance with reduced sensitivity to any particular data split. Throughout this work, we use $k = 5$ (five-fold cross-validation). Kernel bandwidths σ_a are selected via a grid search that maximizes the marginal log-likelihood estimated by five-fold cross-validation.

Each KDE is normalized such that $\int_{-\infty}^{\infty} p_{a,y}(x)\,\mathrm{d}x = 1$, where

$$p_{a,y}(x) = \frac{1}{N_y} \sum_{n=1}^{N_y} \frac{1}{\sqrt{2\pi\sigma_a^2}} \exp\left[\frac{-(x - x_{a,n})^2}{2\sigma_a^2}\right]. \tag{14}$$

Here, N_y is the number of samples for class y and $x_{a,n}$ are the observed values of feature a within that class.

Given an observed feature value x_a^*, we define the probability within a kernel window of half-width $0.5\sigma_a$ centered at x_a^* as

$$\pi_{a,y}(x_a^*) = \int_{x_a^* - 0.5\sigma_a}^{x_a^* + 0.5\sigma_a} p_{a,y}(x)\,\mathrm{d}x. \tag{15}$$

For an environment characterized by m selected features, $\vec{x}^* = (x_1^*, x_2^*, \ldots, x_m^*)$, we compute a class-conditional likelihood score under a naïve Bayes assumption (Fig. 1, Step 4):

$$\mathcal{P}_y(\vec{x}^*) = \prod_{a=1}^{m} \pi_{a,y}(x_a^*). \tag{16}$$

Each environment is assigned the class with the largest likelihood score $\mathcal{P}_y(\vec{x}^*)$.

A further advantage of this probabilistic formulation is explicit out-of-distribution (OOD) detection. To maximize sensitivity, OOD detection is evaluated over the complete descriptor set $\mathcal{D} = \{1, \ldots, M\} \supseteq \mathcal{D}_{\rm cls}$ (see Automatic Feature Selection, above) rather than the m descriptors used for classification alone. For each descriptor $d \in \mathcal{D}$ and class y, we construct $p_{d,y}(x)$ following the same KDE procedure described above, with bandwidth σ_d selected independently via the same five-fold cross-validated grid search. We define an environment as an outlier if $p_{d,y}(x) < P_{\rm cut}$ for all classes y, for any descriptor $d \in \mathcal{D}$. This criterion ensures that the vast majority of in-distribution configurations are retained while enabling detection of novel or anomalous environments, a capability essential for analyzing structural transformations. In this work, we choose $P_{\rm cut} = 5 \times 10^{-4}$. We find that our results are robust up to $P_{\rm cut} = 1 \times 10^{3}$, beyond which outlier configurations are observed during compression/decompression trajectories. This threshold can be adjusted to make the classifier more or less conservative, depending on the application requirements.

D. Training Data and Simulation Details

All configurations were obtained from the Deep Potential (DP) MD trajectories reported in Ref. 8. Two machine-learned potentials were employed, providing complementary representations of water's potential energy surface. The first, DP_SCAN, was trained on electronic-structure data generated with the SCAN density functional [9, 10], a meta-GGA functional known to reproduce many of water's structural and thermodynamic properties. The second, DP_MBpol, was trained on the many-body polarizable (MBpol) potential [11–14], which is parameterized against coupled-cluster reference data and is among the most accurate models of water available. All simulations were performed using LAMMPS (2 Aug 2023) [15] interfaced with DeePMD-kit (v3.0.0) [16, 17], with a timestep of 0.5 fs. Simulations employed a system of 512 water molecules in a cubic box with periodic boundary conditions and the temperature and pressure were controlled via a Nosé–Hoover thermostat and barostat, respectively.

LDA configurations were generated by isobaric quenching from equilibrated liquid water configurations at constant cooling rates of $q_c = 1$ K/ns and $q_c = 10$ K/ns, at 1 bar. Isothermal compression and decompression simulations were conducted starting from LDA configurations at constant rates of $q_p = 300$ bar/ns or $q_p = 3000$ bar/ns. For each pressure and cooling rate, at least three independent simulations were performed to assess statistical variance.

The training and evaluation dataset comprises of 10,000 molecular environments, equally divided between LDA and HDA. Configurations were selected from thermodynamic conditions where phase identity is unambiguous, avoiding the transition region to ensure reliable labels. LDA environments were drawn from isobaric quenches at temperatures at least 30 K below the glass transition and from isothermal compression at pressures at least 3 kbar below the transformation pressure to HDA. HDA environments were taken from compression trajectories at least 3 kbar above the LDA-to-HDA transformation pressure and from decompression trajectories, the latter restricted to densities exceeding that of recovered HDA at 80 K and 1 bar to exclude partially transformed structures. As a benchmark for OOD detection, we curated 1,000 hexagonal ice (Ih) configurations. Ice Ih shares the tetrahedral hydrogen-bond network of LDA but exhibits long-range crystalline order absent in either amorphous phase, making it well-suited for evaluating OOD detection.

S2. BENCHMARKING MODEL PERFORMANCE FOR DP_SCAN ENVIRONMENTS

Martelli and co-workers introduced a supervised neural network (BOO-NN) trained exclusively on bond-orientational order parameters, learning nonlinear mappings from BOO features to phase labels and adopting the traditional notion that orientational symmetry is the key structural discriminator between amorphous phases [3, 4]. DeFever *et al.* proposed a conceptually different strategy with PointNet [18], which bypasses predefined descriptors altogether by learning symmetry-invariant representations directly from atomic coordinates using a permutation-invariant architecture; although highly successful for classifying crystallization and melting pathways, PointNet has not previously been applied to amorphous ices. Boattini and co-workers developed an unsupervised autoencoder-based model [19] that compresses BOO descriptors into a low-dimensional latent space before identifying structural motifs via Gaussian mixture modeling. To enable a supervised comparison, we adapt this method (AE–GMM) by fitting class-conditional multivariate Gaussians to the learned embeddings, capturing nonlinear correlations among BOO features while introducing a latent representation that is less directly interpretable. Collectively, these baselines cover descriptor-based neural networks, coordinate-level representation learning, and latent-space probabilistic modeling, offering a comprehensive set of benchmarks for contextualizing the strengths of our probabilistic classifier.

Here, we provide precisions, recalls, and accuracies of BOO-NN, PointNet, AE-GMM, and the present work on LDA/HDA classification for local atomic environments generated by DP_MBpol (Table S1) and DP_SCAN (Table S2).

TABLE S1: Comparison of model performance for classification of DP_MBpol-generated LDA/HDA environments. Values represent the mean and standard deviation over five-fold cross-validation.

Model	HDA-Precision	HDA-Recall	LDA-Precision	LDA-Recall	Accuracy
BOO-NN	0.997 ± 0.001	0.997 ± 0.002	0.997 ± 0.002	0.997 ± 0.001	0.997 ± 0.001
PointNet	0.937 ± 0.008	0.988 ± 0.003	0.988 ± 0.003	0.934 ± 0.008	0.961 ± 0.003
AE-GMM	0.938 ± 0.008	0.982 ± 0.003	0.983 ± 0.003	0.935 ± 0.008	0.959 ± 0.003
Present Work (BOO)	0.997 ± 0.001	0.968 ± 0.010	0.967 ± 0.010	0.997 ± 0.001	0.982 ± 0.005
Present Work (ACSF)	0.973 ± 0.012	0.988 ± 0.009	0.987 ± 0.010	0.972 ± 0.013	0.980 ± 0.003
Present Work (BOO+ACSF)	0.996 ± 0.001	0.989 ± 0.003	0.989 ± 0.003	0.996 ± 0.001	0.993 ± 0.001

TABLE S2: Comparison of model performance for classification of DP_SCAN-generated LDA/HDA environments. Values represent the mean and standard deviation over five-fold cross-validation.

Model	HDA-Precision	HDA-Recall	LDA-Precision	LDA-Recall	Accuracy
BOO-NN	0.947 ± 0.007	0.997 ± 0.001	0.997 ± 0.001	0.945 ± 0.007	0.971 ± 0.003
PointNet	0.937 ± 0.008	0.988 ± 0.003	0.988 ± 0.003	0.934 ± 0.008	0.961 ± 0.003
AE-GMM	0.938 ± 0.008	0.982 ± 0.003	0.983 ± 0.003	0.935 ± 0.008	0.959 ± 0.003
Present Work (BOO)	0.951 ± 0.006	0.977 ± 0.008	0.978 ± 0.008	0.950 ± 0.005	0.964 ± 0.006
Present Work (ACSF)	0.935 ± 0.010	0.997 ± 0.001	0.998 ± 0.001	0.931 ± 0.010	0.964 ± 0.005
Present Work (BOO+ACSF)	0.948 ± 0.006	0.987 ± 0.004	0.988 ± 0.004	0.945 ± 0.005	0.967 ± 0.004

Performance metrics are lower for classifying environments generated by DP_SCAN than they are for DP_MBpol. Table S1 shows that accuracies for some models are greater than 0.99 for DP_MBpol environments, while here all accuracies are roughly between 0.96-0.97 for DP_SCAN environments. The relative performance of the models, however, is similar; BOO-NN and our work perform best, followed by PointNet and AE-GMM. These results agree with what is observed for DP_MBpol environments: our work maintains comparable accuracy to deep learning methods while adopting a physically interpretable classification scheme. Interestingly, BOO-NN, AE-GMM, and our work show higher HDA recall and LDA precision than HDA precision and LDA recall. This indicates that these models are liberal in their assignment of HDA but conservative in their assignment of LDA, suggesting a relationship between HDA and LDA structures for DP_SCAN not present in DP_MBpol.

S3. OUT-OF-DISTRIBUTION PREDICTIONS FOR DP_SCAN AND DP_MBPOL ENVIRONMENTS

In the main text, Section 2.1. we analyze the behavior of BOO-NN, PointNet, AE-GMM, and this work when making predictions on out-of-distribution atomic environments. Specifically, models are trained on HDA and LDA atomic environments, but are used to predict the labels of hexagonal ice (Ih) atomic environments. In the main text, we only perform this analysis for environments generated by DP_MBpol. Here, we report the same results for DP_SCAN configurations.

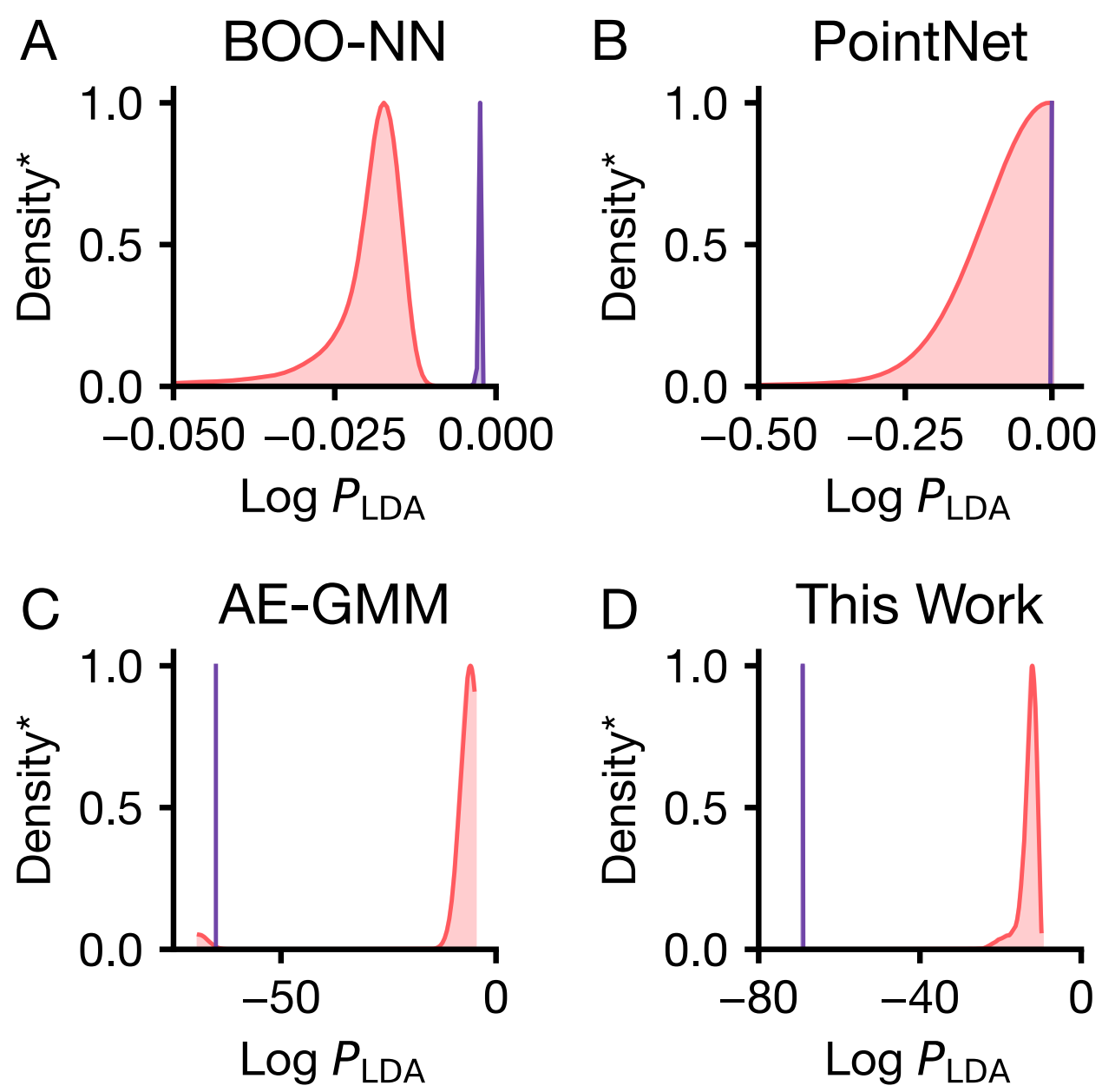

FIG. S1: Predicted LDA log probabilities from (A) BOO-NN, (B) PointNet, (C) AE-GMM, and (D) this work applied to DP_SCAN-generated environments of LDA and hexagonal ice (Ih). Distributions for true LDA configurations are shown in red, while distributions for Ih configurations are shown in purple. Each density is normalized so that its maximum value is 1.0.

As observed in the main text, BOO-NN (Fig. S1A) and PointNet (Fig. S1B) struggle to separate LDA and Ih distributions, and these models confidently label Ih environments as LDA. Accordingly, the present work is capable of accurately distinguishing Ih environments as outliers from those on which the model was trained (*i.e.*, LDA and HDA), as shown by the separation between the distributions in Figure S1D. Interestingly, while AE-GMM (Fig. S1C) was capable of detecting outliers for DP_MBpol environments, this model struggles to do so for configurations generated by DP_SCAN. Such a result demonstrates the utility of considering all available features, as is done in our work, relative to density estimation on reduced feature spaces, as is done by AE-GMM.

S4. OUT-OF-DISTRIBUTION PREDICTION FOR ALTERNATIVE PHASES OF ICE

Our outlier detection method identifies local atomic environments that differ from the LDA/HDA training distribution along any structural dimension. To evaluate how the choice of features affects out-of-distribution (OOD) accuracy, we compare two approaches applied to an expanded dataset of ice polymorphs obtained from the datasets which were used in the training of the DP_SCAN and DP_MBpol models [10, 14]

The first approach uses only the five selected features most informative for LDA/HDA classification. As discussed in the main text, this results in a sparse, interpretable model whose features directly reflect what distinguishes the two amorphous states from one another. The second approach includes all available features after removing pairwise-correlated descriptors (Pearson $|r| > 0.8$), retaining a diverse, uncorrelated subset of the full 120-dimensional descriptor space. This is not equivalent to using all 120 descriptors simultaneously. Including strongly correlated features in a naive Bayes product model would double-count shared structural information and degrade OOD sensitivity.

TABLE S3: Accuracy of classifiers that use different numbers of features for OOD classification. Models are used to classify structures of different ice phases generated by the DP_MBpol and DP_SCAN water models. Models can either use five features relevant to LDA/HDA classification ($n = 5$) or use all uncorrelated provided structural descriptors ($n = 120$).

Phase	DP_MBpol		DP_SCAN	
	$n = 5$	$n = 120$	$n = 5$	$n = 120$
Ice Ih	1.000	1.000	1.000	1.000
Ice Ic	1.000	1.000	0.998	0.998
Ice II	0.000	0.122	0.003	0.378
Ice III	0.009	0.933	0.001	1.000
Ice IV	0.012	0.033	0.048	0.071
Ice V	0.003	0.872	0.064	0.998
Ice VI	0.024	0.134	0.087	0.250
Ice VII	1.000	1.000	1.000	1.000
Ice VIII	1.000	1.000	1.000	1.000
Ice IX	0.005	0.993	0.000	1.000
Ice XI	0.993	0.998	1.000	1.000
Ice XII	0.062	0.991	0.30	1.000
Ice XIII	0.006	0.763	0.052	0.980
Ice XIV	0.031	0.987	0.312	1.000
Ice XV	0.061	0.214	0.264	0.470

Table S3 shows OOD detection rates for both approaches across 14 ice polymorphs. Using the full uncorrelated descriptor set outperforms the five-feature model in most cases, with several phases moving from near-zero to near-complete detection. This indicates that the combined ACSF and BOO descriptor set contains structural information sufficient to distinguish the majority of ice polymorphs from amorphous ice. A subset of high-pressure phases (e.g., Ice II, IV, VI, and XV) remain only partially distinguishable from LDA/HDA even when using the full descriptor set.

This residual ambiguity is a consequence of the descriptor selection procedure used here, rather than the local environment representation itself. At the level of individual atomic neighborhoods, some high-pressure ice phases share geometric features with LDA/HDA that are not fully resolved by the descriptors identified that optimally distinguish LDA and HDA. This does not indicate that these crystalline phases are physically present within computational LDA or HDA. Independent molecular dynamics studies of LDA and HDA have found no evidence of ice-like domains in either amorphous phase [20]. Taken together, these results suggest that the observed classifier ambiguity for certain high-pressure ices arises from local geometric similarity between amorphous and crystalline packing motifs along the LDA/HDA classification axis, rather than from partially crystallized regions embedded within the amorphous structures. Distinguishing such phases would be possible if a classifier were directly trained to resolve the amorphous states from the crystalline ones.

S5. SENSITIVITY OF STRUCTURAL PATHWAYS TO SELECTED FEATURE SPACES

In the main text, Section 2.6 analyzes how the evolution of average atomic environments proceeds during compressions and decompressions across a range of trajectories at different temperatures. We show these evolutions for DP_MBpol and DP_SCAN for two different feature spaces. Here, we show these evolutions for 25 feature spaces comprised of each pair of features selected by our model to be most informative for accurate LDA/HDA classification.

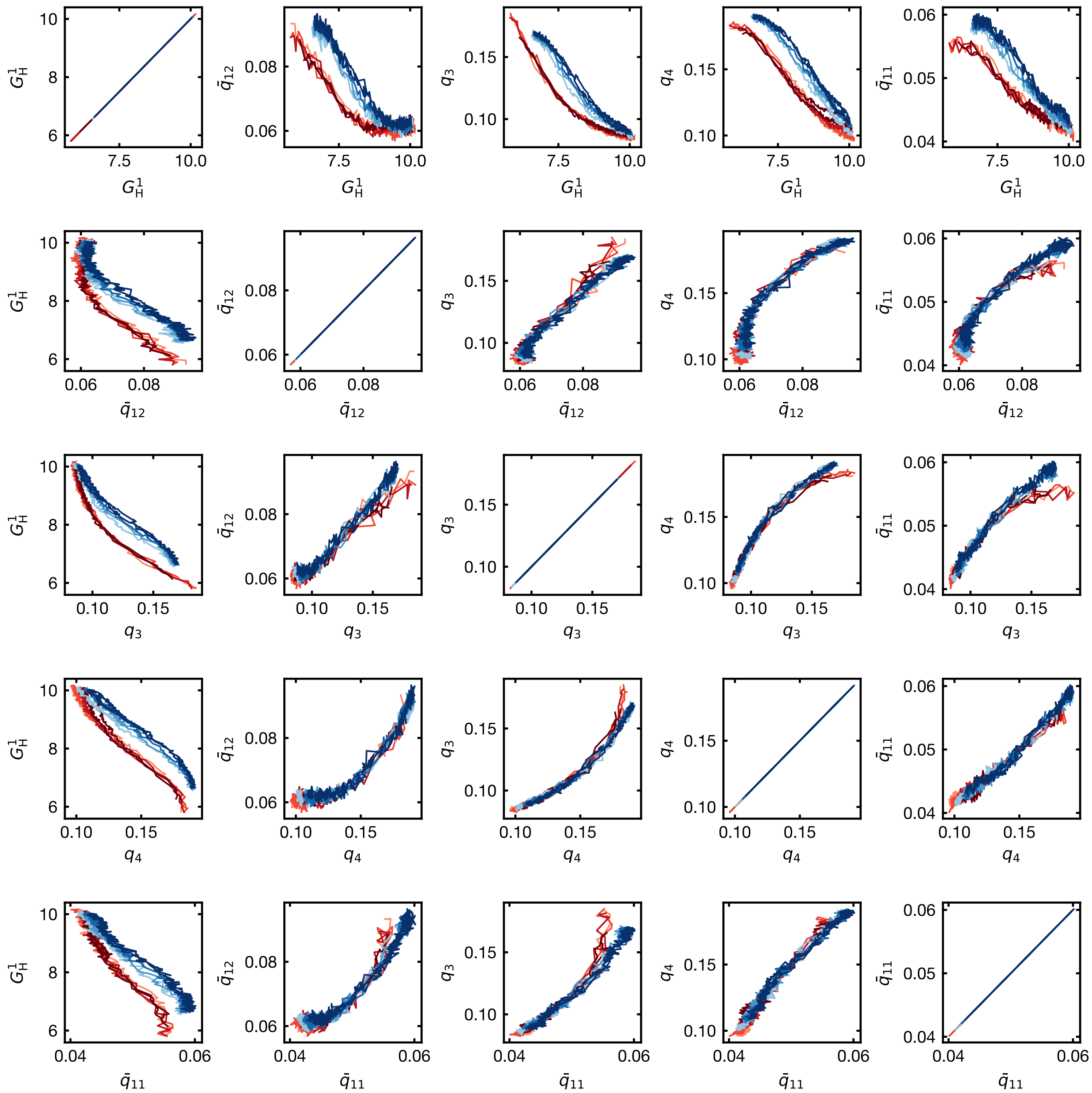

FIG. S2: Evolution of average local environment descriptors during compressions (blue) and decompressions (red) at temperatures from 60 K to 140 K for the DP_MBpol force field. Descriptors are plotted in feature spaces for every pair of features selected by our model for LDA/HDA classification. Darker red/blue shades correspond to lower temperatures.

Results from Figures S2 and S3 corroborate the results discussed in Section 2.6. Namely, we observe a hysteresis in environment pathways when considering feature spaces that consist of an ACSF and BOO, but this separation is not visible when visualizing pairs of BOOs. Therefore, it is necessary to consider both radial and spherical information to identify differences in the structural pathways taken by compressions and decompressions—an observation that would go unnoticed if we relied solely on the BOOs employed in previous studies.

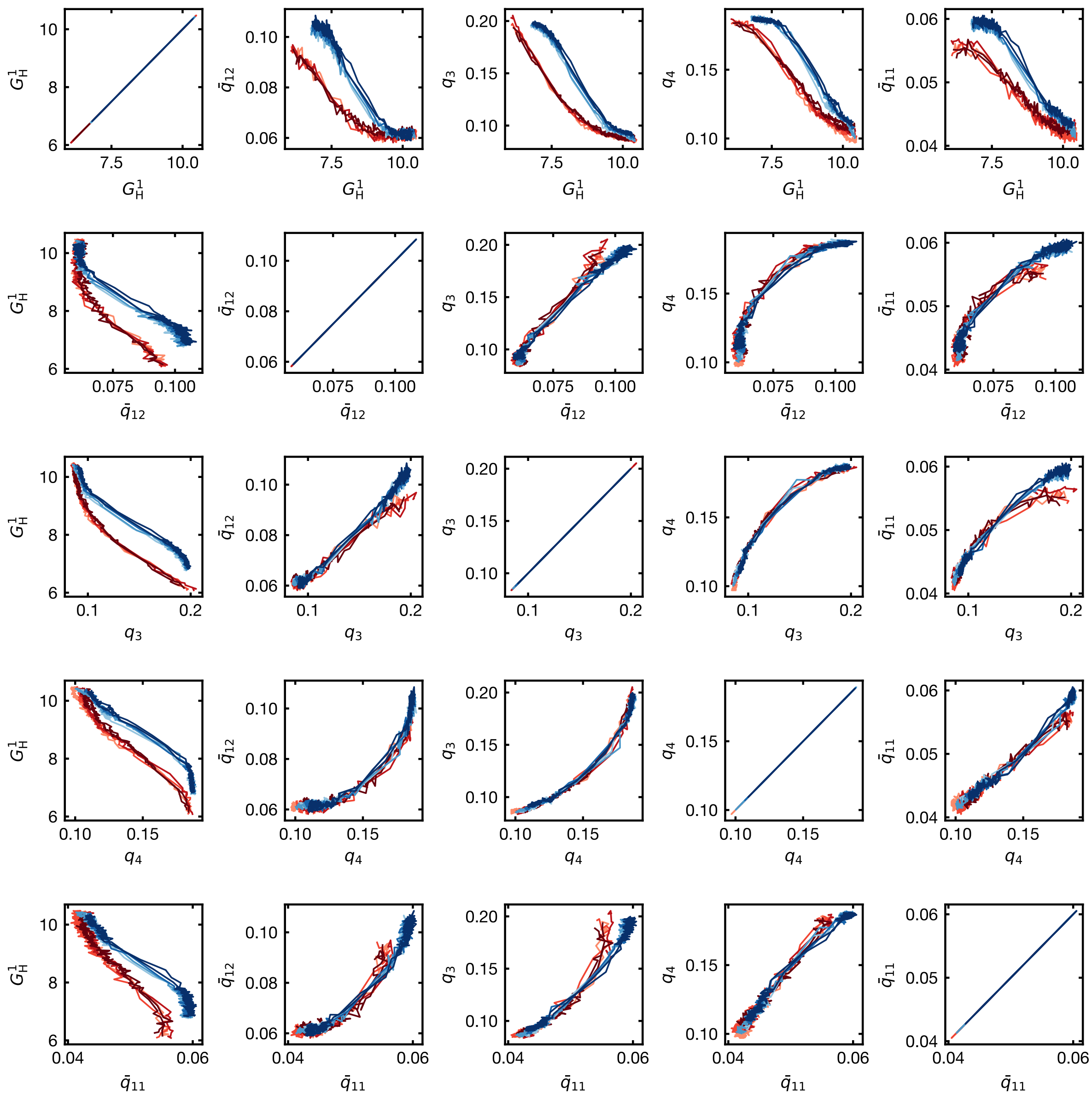

FIG. S3: Evolution of average local environment descriptors during compressions (blue) and decompressions (red) at temperatures from 60 K to 140 K for the DP_SCAN force field. Descriptors are plotted in feature spaces for every pair of features selected by our model for LDA/HDA classification. Darker red/blue shades correspond to lower temperatures.

S6. DIFFERENCES BETWEEN DP_SCAN- AND DP_MBPOL-GENERATED HDA ENVIRONMENTS

In the main text, Section 2.7 analyzes structural differences between LDA environments generated by DP_SCAN and DP_MBpol. Here, we consider structural differences between HDA environments generated by the same two force fields. Our classifier can distinguish between DP_SCAN and DP_MBpol HDA environments with an accuracy of 64.3 ± 0.03%, indicating that there are structural differences (albeit less than LDA) present in these environments. We consider how distributions of DP_SCAN and DP_MBpol environments vary along a select set of structural descriptors with high mutual information for DP_SCAN/DP_MBpol classification, which we visualize in Figure S4.

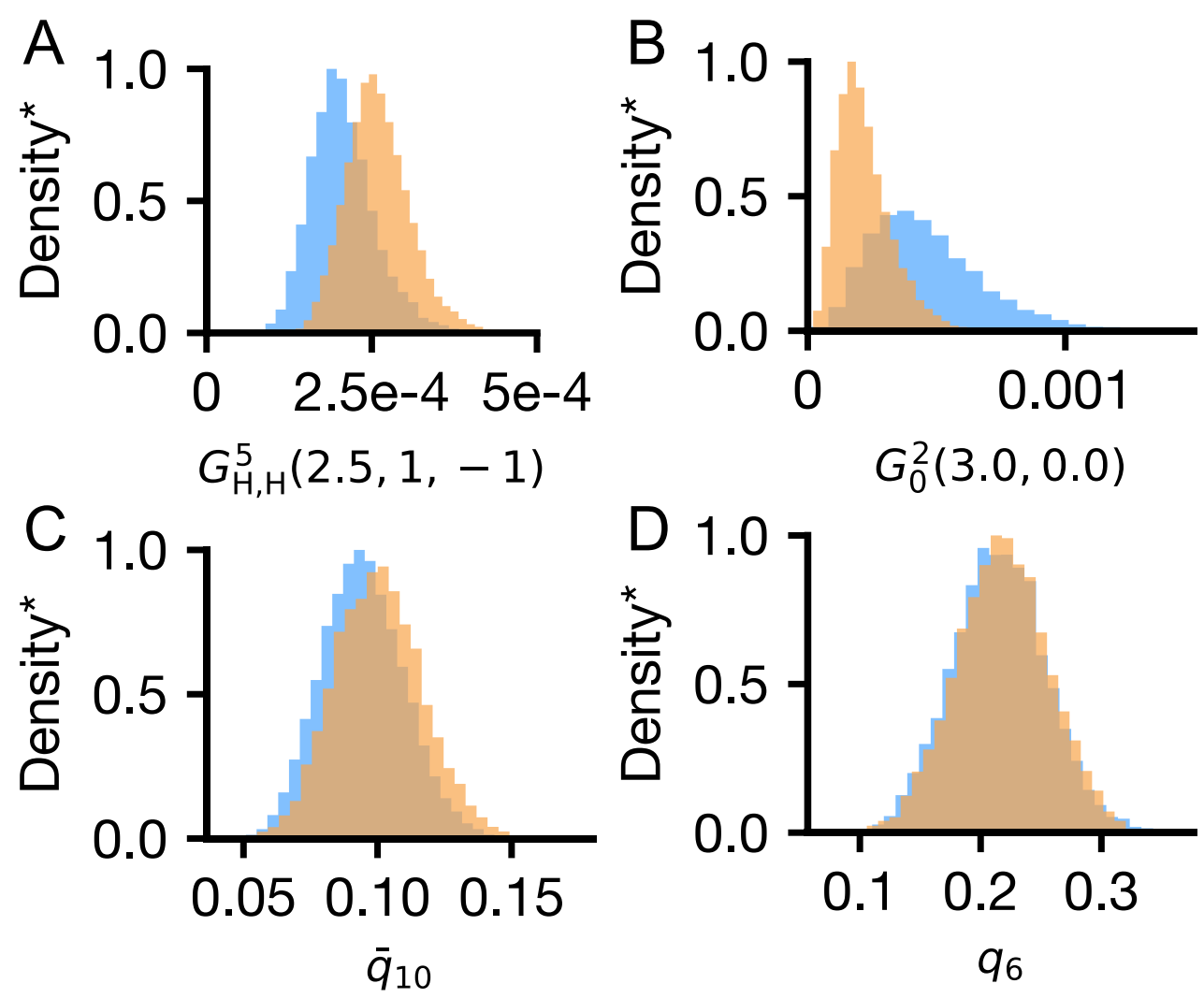

FIG. S4: Differences between local environments of HDA generated from DP_SCAN and DP_MBpol. (A–D) Distributions of select ACSF and BOO descriptors for HDA generated by each model. All probability densities are normalized such that the maximum value of either distribution is 1.0 (denoted as Density*). The blue and orange colors represent the DP_SCAN and DP_MBpol models, respectively.

Figures S4A and S4B show distributions of DP_SCAN and DP_MBpol environments along the two most informative features identified by our classifier. One can clearly observe differences in the two distributions, suggesting that (similar to LDA environments) DP_SCAN and DP_MBpol disagree most in their reproduction of interatomic distances. This is most directly observed when considering the second descriptor, $G^2_{\mathrm{O}}(3.0, 0.0)$, where higher values indicate reduced oxygen–oxygen pairwise distances. Figures S4C and S4D show distributions of DP_SCAN and DP_MBpol environments along the two most informative BOOs. HDA environments appear to have similar distributions along these descriptors, indicating that DP_SCAN and DP_MBpol predict similar spherical symmetry in HDA environments.

[1] J. Behler, Atom-centered symmetry functions for constructing high-dimensional neural network potentials, J. Chem. Phys. **134**, 074106 (2011).
[2] P. J. Steinhardt, D. R. Nelson, and M. Ronchetti, Bond-orientational order in liquids and glasses, Phys. Rev. B **28**, 784 (1983).
[3] F. Martelli, F. Leoni, F. Sciortino, and J. Russo, Connection between liquid and non-crystalline solid phases in water, J. Chem. Phys. **153**, 104503 (2020).
[4] Z. Faure Beaulieu, V. L. Deringer, and F. Martelli, High-dimensional order parameters and neural network classifiers applied to amorphous ices, J. Chem. Phys. **160**, 081101 (2024).
[5] W. Lechner and C. Dellago, Accurate determination of crystal structures based on averaged local bond order parameters, J. Chem. Phys. **129**, 114707 (2008).
[6] E. D. Donkor, A. Offei-Danso, A. Rodriguez, F. Sciortino, and A. Hassanali, Beyond local structures in critical supercooled water through unsupervised learning, J. Phys. Chem. Lett. **15**, 3996 (2024).
[7] C. E. Shannon, A mathematical theory of communication, The Bell System Technical Journal **27**, 379 (1948).
[8] R. J. Szukalo, N. Giovambattista, and P. G. Debenedetti, Computational investigation of water glasses using machine-learning potentials, Proc. Natl. Acad. Sci. U.S.A. **122**, e2509609122 (2025).
[9] J. Sun, A. Ruzsinszky, and J. P. Perdew, Strongly constrained and appropriately normed semilocal density functional, Phys. Rev. Lett. **115**, 036402 (2015).
[10] L. Zhang, H. Wang, R. Car, and W. E, Phase diagram of a deep potential water model, Phys. Rev. Lett. **126**, 236001 (2021).
[11] V. Babin, C. Leforestier, and F. Paesani, Development of a "first principles" water potential with flexible monomers: Dimer potential energy surface, VRT spectrum, and second virial coefficient, J. Chem. Theory Comput. **9**, 5395 (2013).
[12] V. Babin, G. R. Medders, and F. Paesani, Development of a "first principles" water potential with flexible monomers. ii: Trimer potential energy surface, third virial coefficient, and small clusters, J. Chem. Theory Comput. **10**, 1599 (2014).
[13] G. R. Medders, V. Babin, and F. Paesani, Development of a "first-principles" water potential with flexible monomers. iii. liquid phase properties, J. Chem. Theory Comput. **10**, 2906 (2014).
[14] S. L. Bore and F. Paesani, Realistic phase diagram of water from "first principles" data-driven quantum simulations, Nat. Comm. **14**, 3349 (2023).
[15] A. P. Thompson, H. M. Aktulga, R. Berger, D. S. Bolintineanu, W. M. Brown, P. S. Crozier, P. J. In't Veld, A. Kohlmeyer, S. G. Moore, T. D. Nguyen, et al., LAMMPS-a flexible simulation tool for particle-based materials modeling at the atomic, meso, and continuum scales, Comput. Phys. Commun. **271**, 108171 (2022).
[16] L. Zhang, J. Han, H. Wang, R. Car, and W. E, Deep potential molecular dynamics: a scalable model with the accuracy of quantum mechanics, Phys. Rev. Lett. **120**, 143001 (2018).
[17] H. Wang, L. Zhang, J. Han, and E. Weinan, DeePMD-kit: A deep learning package for many-body potential energy representation and molecular dynamics, Comput. Phys. Commun. **228**, 178 (2018).
[18] R. S. DeFever, C. Targonski, S. W. Hall, M. C. Smith, and S. Sarupria, A generalized deep learning approach for local structure identification in molecular simulations, Chem. **10**, 7503 (2019).
[19] E. Boattini, M. Dijkstra, and L. Filion, Unsupervised learning for local structure detection in colloidal systems, J. Chem. Phys. **151**, 154901 (2019).
[20] F. Martelli, N. Giovambattista, S. Torquato, and R. Car, Searching for crystal-ice domains in amorphous ices, Phys. Rev. Mater. **2**, 075601 (2018).
