# Supplementary material for: A local structural basis to distinguish amorphous ices
Source: Nat Commun. 2026 Sep 12;17:10754. doi: 10.1038/s41467-026-77279-5 (PMC13661517; doi:10.1038/s41467-026-77279-5)
Supplement: Supplementary file 2 [file 41467_2026_77279_MOESM2_ESM.pdf]

nature portfolio

Peer Review File

A Local Structural Basis to Resolve Amorphous Ices

Corresponding Author: Professor Michael Webb

This file contains all reviewer reports in order by version, followed by all author rebuttals in order by version.

Version 0:

Reviewer comments:

Reviewer #1

(Remarks to the Author)
We expect distinct macroscopic thermodynamic states to be characterized by equally distinct microscopic states, and number of methods have been developed that distinguish between crystals, such as FCC, HPC BCC and the liquid in terms of local atomic/molecular environments. However, identifying local environments that distinguish between distinct amorphous states remains a significant challenge. The current manuscript introduces an interpretable probabilistic classification method that uses a mutual information procedure to rank the effectiveness of descriptors and assigns probabilities that a given local environment belongs to a particular macroscopic state.
The method is applied to the study of the transformation between the low density (LDA) and high density (HDA) amorphous ices of water, using both atom-centered symmetry functions (ACSF) that capture the local radial density distribution and Steinhardt based bond orientational order (BOO) parameters, where all quantities are measured over the nearest 16 neighbours.

The work demonstrates that the method can distinguish between LDA and HDA states and that the transition occurs in a first order like way, without proceeding through intermediate states, during both compression and decompression. In particular, both AFSF and BOO are needed to distinguishes states but the ACSF, provide the most important measure. It is also interesting to note that the approach identifies differences in the nature of the microscopic LDA phases for different intermolecular potentials.

The manuscript is well written, and the results support the conclusions. It also directly addresses a key challenge regarding the nature of LDA-HDA transition in water. The method also has the potential to address wider applications for the identification of local structure in other glassy materials. As such I can recommend publication in Nature communications essentially as is.

I have a couple of questions the authors could address.
1. The glassy nature of the amorphous ices may significantly narrow the distribution of local states compared to say an equilibrium liquid states that may be connected to the amorphous ices, i.e have greater vibrational variability. Is it likely the accuracy of the method persists in liquid states.
2. I found it a little difficult to follow the behaviour outlined in Figure 4(F). The same information plotted in 2D form in fig. S2 and S3 was easier to identify. This is is just a minor point but it might be useful to also reference the figures in the SI.

(Remarks on code availability)
The code is well organized and commented. Data set are also provided for analysis.

Reviewer #2

(Remarks to the Author)
In the paper entitled “A Local Structural Basis to Resolve Amorphous Structures: The Case of Amorphous Ices” (Gallagher, et al.) the authors address one of the most challenging problem of discriminating the microscopic structures of low-density and high-density amorphous (LDA and HDA) ices, which are commonly viewed as vitrified low-density and high-density states of metastable supercooled liquid water. They answer, in particular, the question of which molecular scale the local environments of LDA and HAD differ.

This is a high-quality computational study. The authors use a sophisticated probabilistic framework applied to molecular

simulations of water to detect structural configurations of LDA and HAD states. This probabilistic framework is applicable to any system in which different amorphous or disordered phases lack obvious microscopic distinguishing features.
LDA exhibits an open, tetrahedrally ordered network, whereas HDA is denser and more disordered. Previous studies of supercooled liquid water suggest that distinguishing low-and high-density environments requires correlations extending beyond the first coordination shell, however the question whether the nonlocality reflects fundamental structural physics has not been assessed in the previous studies.

The most interesting and surprising result of this new study is that the structural identity is fundamentally defined within the first coordination shell. The authors have found that interstitial hydrogen density dominates with the phase identity being encoded within the first coordination shell. Analysis of transformation trajectories reveals no intermediate structures. The transition between the two states occurs through redistribution of LDA-like and HDA-like environments. The local hydrogen density exposes structural hysteresis between compression and decompression pathways that is entirely invisible to previously introduced popular "orientational order parameters". These results are new and of broad interest for scientific community.

Overall, I have a very positive impression of this work that deserves to be published in Nature Communications. However, I have recommendations to the authors, which can enhance broader impacts of the paper.

It would be useful (in a broader scientific context) to see a brief discussion of a link with generic phenomenology of polyamorphism (see, for example, Anisimov, et al. "Thermodynamics of Fluid Polyamorphism", Phys. Rev. X 8, 011004, 2018). In this approach, the equilibrium distribution between the two structures of supercool water is described through the fraction of interconversion, a phenomenological order parameter. Logarithm of the equilibrium constant is viewed as the ordering field conjugate to the order parameter. This phenomenology was successfully applied to the description of the properties of supercooled water and several water-like models, such as TIP4P/2005 and S2.

In a relatively recent study (Singh, et al., "Two-State Thermodynamics and the Possibility of a Liquid-Liquid Phase Transition in Supercooled TIP4P/2005", J. Chem. Phys., 144, 144504, 2016) the authors compared the phenomenological order parameter (fraction of interconversion) with hypothesized microscopic features based on two different criteria: distance to the fifth nearest neighbor (d5) and local structure index (LSI). The parameter d5 contains information about the local structure up to the first coordination shell (3.5 Å) only. Comparison between the prediction based on d5 and the phenomenological EOS revealed near perfect agreement. Structural information beyond the first shell was presumably obtained through LSI. Interestingly, the application of LSI did not help in comparison with the phenomenological EOS. In fact, it demonstrates significant disagreement with the interconversion fraction of two-state thermodynamics, which, in my opinion, resonates with the major result of the paper under review.

In summary I conclude that the paper of Gallagher, et al. can be published in Nature Communications but it requires a minor revision.

(Remarks on code availability)

Reviewer #3

(Remarks to the Author)
Gallagher and co-workers present an analysis of simulated data of amorphous ices. In their work, local structural information is encoded as a structural descriptor typical for machine learning. Based on classification with a drastically reduced version of the descriptor, the authors report that the only two amorphous local structural classes, low-density (LDA) and high-density (HDA), can be found during the pressure-induced phase transition in between them. The authors conclude the simulated trajectories show a first order phase transition with hysteresis, and emphasize the role of an interstitial water molecule in the first solvation shell.

I have no doubt that a full ACSF, or some of the numerous other descriptors, would able to do such a classification after a suitable model selection procedure. This work concludes that it suffices to consider a small subset of interpretable features thereof. The authors base their analysis on descriptor features, selected without human bias, that can still be converted back to human-interpretable information. This aspect adds notable general value to the work. In my opinion, the authors correctly identify that the target of the investigation dictates which descriptor performs best, and that the obtainable information is also dictated by this choice.

The work is timely considering the ongoing discussion about amorphous phases of ice. I find the work methodologically interesting as well. However, some of the following points necessitate a major revision, before I can personally recommend publication.

1. The results of the work rely on classifying OOD correctly. For training, the authors use data from the extrema of the pressure range where no ambiguity exists for LDA/HDA. However, the training data does not contain OOD structures, the hidden third class of the prediction output. While a ACSF descriptor of a high number of features would probably encode all relevant structural information, the authors exclude descriptor features based on data of these two phases (HDA/LDA) alone. This raises a question if the dropped features would trigger OOD, if included in the analysis procedure.

2. The same cross validation is used for training and for evaluation of performance. The orthodox way to study performance is to split the data to a separate training and test parts to avoid leakage of information. If any choice or step is based on CV score for training, then information leaks from test to training.

3. Since the work relies on LDA/HDA/OOD classification, a notable fraction of the test set should consist of OOD structures of relevant local densities. In particular this calls for high pressure ice phases and the liquid structures at high pressures, that are reasonably close in the local configuration space. As a consequence of the present choice, the reported accuracy 99.3 % is skewed to the optimistic side due to selection bias toward extreme LDA/HDA cases. More critically, evidence for performance is largely missing for the OOD structures, apart from a test for 1000 structures of hexagonal ice. While the spirit of this test is to be appreciated, one has to remember that the structural descriptor vector has been heavily truncated based on data for only LDA and HDA. I think a study to verify performance could be doable based on published structures of relevant local densities. In my opinion, the drop in performance observed for classification of structures from the other studied force field already underlines this point.

4. Given the spatial limitations of the selected features, how would the authors even detect a change further from the origin? In other words, the question setting limits the possible conclusions to the first solvation shell, without a test showing that such a cutoff would be suitable for the studied question. This point could be addressed by slight re-wording in the manuscript.

5. The caption for figure 4 F does not explain the plot sufficiently. While the pair of curves up in GH1 are clear, the description of the curves with lower GH1 feature values is absent. From the text on page 7 ("when GH1 is replaced by q4") I deduce that these curves represent a projection on the q3-q4 plane.

6. Is the "temperature" cited for curves of figure 4F evaluated for a local environment, or are these results from separate MD runs?

7. On page 6, right column "structurally distinct regions of configuration space" might need "local", as the analysis is carried out for maximum of 17 water molecules.

-minor points in the supplement-

8. SI section A, phrasing: A molecule can not be classified as LDA/HDA. A sufficiently large local environment may be.

9. SI page 5: a broken reference.

10. SI: MD lacks details such as the timestep, the system size, the ensemble and preparation of the initial state of the system. I assume a constant number of molecules with a constant energy and a varying reference pressure.

(Remarks on code availability)

Version 1:

Reviewer comments:

Reviewer #1

(Remarks to the Author)
My concerns have been addressed. I believe the manuscript is now suitable for publication.

(Remarks on code availability)

Reviewer #2

(Remarks to the Author)

(Remarks on code availability)
I recommend publishing the revised version as is.

Reviewer #3

(Remarks to the Author)
The authors have satisfactorily addressed my comments and questions. Indeed, the local structural hysteresis must persist regardless of whether a complete descriptor vector is employed, provided that the three identified features are included in the analysis. Moreover, I believe it is important to keep the possibility of out-of-distribution effects in mind to avoid overconfidence when identifying structural classes.

The work by Gallagher and co-workers has clear merits, and I recommend publication.

Minor comment: On page 9 (right column), there is a placeholder "XXX" that should be replaced with the corresponding numerical value.

(Remarks on code availability)

Open Access This Peer Review File is licensed under a Creative Commons Attribution 4.0 International License, which permits use, sharing, adaptation, distribution and reproduction in any medium or format, as long as you give appropriate credit to the original author(s) and the source, provide a link to the Creative Commons license, and indicate if changes were made.
In cases where reviewers are anonymous, credit should be given to 'Anonymous Referee' and the source.
The images or other third party material in this Peer Review File are included in the article's Creative Commons license, unless indicated otherwise in a credit line to the material. If material is not included in the article's Creative Commons license and your intended use is not permitted by statutory regulation or exceeds the permitted use, you will need to obtain permission directly from the copyright holder.
To view a copy of this license, visit https://creativecommons.org/licenses/by/4.0/

Response to Reviewers

A Local Structural Basis to Distinguish Amorphous Ices

Gallagher, Szukalo, Giovambattista, Debenedetti, and Webb

Nature Communications — Manuscript ID: NCOMMS-26-026965-T

July 5, 2026

We thank the three reviewers for their careful and thorough reading of our manuscript and for their constructive comments, which have strengthened the work. We have carefully addressed all comments and have revised the manuscript accordingly. Below, we provide detailed responses to each reviewer comment. Our narrative responses are *italicized*, and any changes to the manuscript are highlighted by red text. A marked-up version of the revised manuscript is included with this response.

1 Reviewer 1

Reviewer Comment:

We expect distinct macroscopic thermodynamic states to be characterized by equally distinct microscopic states, and number of methods have been developed that distinguish between crystals, such as FCC, HPC, BCC and the liquid in terms of local atomic/molecular environments. However, identifying local environments that distinguish between distinct amorphous states remains a significant challenge. The current manuscript introduces an interpretable probabilistic classification method that uses a mutual information procedure to rank the effectiveness of descriptors and assigns probabilities that a given local environment belongs to a particular macroscopic state. The method is applied to the study of the transformation between the low density (LDA) and high density (HDA) amorphous ices of water, using both atom-centered symmetry functions (ACSF) that capture the local radial density distribution and Steinhardt based bond orientational order (BOO) parameters, where all quantities are measured over the nearest 16 neighbours.

The work demonstrates that the method can distinguish between LDA and HDA states and that the transition occurs in a first order like way, without proceeding through intermediate states, during both compression and decompression. In particular, both AFSF and BOO are needed to distinguishes states but the ACSF, provide the most important measure. It is also interesting to note that the approach identifies differences in the nature of the microscopic LDA phases for different intermolecular potentials.

The manuscript is well written, and the results support the conclusions. It also directly addresses a key challenge regarding the nature of LDA-HDA transition in water. The method also has the potential to address wider applications for the identification of local structure in other glassy materials. As such I can recommend publication in Nature communications essentially as is.

Author Response:

We are very grateful to Reviewer 1 for taking the time to review the manuscript and provide such a positive assessment and endorsement for publication. We address their two specific questions below.

1.1 Comments

Reviewer Comment:

The glassy nature of the amorphous ices may significantly narrow the distribution of local states compared to say an equilibrium liquid state that may be connected to the amorphous ices, i.e., have greater vibrational variability. Is it likely the accuracy of the method persists in liquid states?

Author Response:

The reviewer raises an insightful point. The arrested nature of the glass confines molecules to a restricted region of local configuration space around their inherent structures, producing narrower descriptor distributions than those of an equilibrium liquid. In the liquid, thermal fluctuations broaden these distributions and increase overlap between low- and high-density environments, potentially necessitating a larger or a different set of descriptors to achieve comparable accuracy. Nevertheless, we believe the probabilistic framework itself is well-suited to this setting. Given sufficiently informative descriptors, the mutual-information selection procedure would identify the relevant ones, and the density-estimation-based classifier would construct appropriate class boundaries. Applying this framework to classify low- and high-density liquid (LDL/HDL) environments as one approaches the liquid–liquid critical point is a natural and compelling direction for future work. We have added the following to the Implications section of the revised manuscript:

> ***Page 8, Implications****:*
> *This state-dependence also suggests that extending the framework to equilibrium liquid environments, where broader thermal fluctuations are expected to increase descriptor overlap, may require a larger or different descriptor set. Classification of LDL and HDL environments as the liquid–liquid critical point is approached represents a compelling direction for future work.*

Reviewer Comment:

I found it a little difficult to follow the behaviour outlined in Figure 4(F). The same information plotted in 2D form in Figures S2 and S3 was easier to identify. This is just a minor point but it might be useful to also reference the figures in the SI.

Author Response:

We thank the reviewer for this suggestion. We have clarified panel F of the caption to explicitly describe both sets of curves shown in the 3D plot and reference the 2D projections in the SI.

> ***Figure 4 Caption:***
> ***(F)*** *Mean local-environment descriptors (q_3, q_4, G^1_{H}) along compression (blue) and decompression (red) pathways for separate isothermal MD simulations at temperatures from 60 to 140 K. The upper set of curves shows trajectories in the full q_3–q_4–G^1_{H} space; the lower set shows the same trajectories projected onto the q_3–q_4 plane. Stars mark transition points; darker shades correspond to lower temperatures. See SI Figures S2 and S3 for 2D projections.*

2 Reviewer 2

Reviewer Comment:

In the paper entitled "A Local Structural Basis to Resolve Amorphous Structures: The Case of Amorphous Ices" (Gallagher, et al.) the authors address one of the most challenging problem of discriminating the microscopic structures of low-density and high-density amorphous (LDA and HDA) ices, which are commonly viewed as vitrified low-density and high-density states of metastable supercooled liquid water. They answer, in particular, the question of which molecular scale the local environments of LDA and HDA differ.

This is a high-quality computational study. The authors use a sophisticated probabilistic framework applied to molecular simulations of water to detect structural configurations of LDA and HAD states. This probabilistic framework is applicable to any system in which different amorphous or disordered phases lack obvious microscopic distinguishing features. LDA exhibits an open, tetrahedrally ordered network, whereas HDA is denser and more disordered. Previous studies of supercooled liquid water suggest that distinguishing low-and high-density environments requires correlations extending beyond the first coordination shell, however the question whether the nonlocality reflects fundamental structural physics has not been assessed in the previous studies.

The most interesting and surprising result of this new study is that the structural identity is fundamentally defined within the first coordination shell. The authors have found that interstitial hydrogen density dominates with the phase identity being encoded within the first coordination shell. Analysis of transformation trajectories reveals no intermediate structures. The transition between the two states occurs through redistribution of LDA-like and HDA-like environments. The local hydrogen density exposes structural hysteresis between compression and decompression pathways that is entirely invisible to previously introduced popular "orientational order parameters". These results are new and of broad interest for scientific community.

Overall, I have a very positive impression of this work that deserves to be published in Nature Communications. However, I have recommendations to the authors, which can enhance broader impacts of the paper.

Author Response:

We thank Reviewer 2 for their time in providing a thorough assessment of the work. Their positive commentary is especially gratifying. We address their specific recommendations below.

2.1 Comments

Reviewer Comment:

It would be useful (in a broader scientific context) to see a brief discussion of a link with generic phenomenology of polyamorphism (see, for example, Anisimov et al., "Thermodynamics of Fluid Polyamorphism," *Phys. Rev. X* **8**, 011004, 2018). In this approach, the equilibrium distribution between the two structures of supercooled water is described through the fraction of interconversion, a phenomenological order parameter. Logarithm of the equilibrium constant is viewed as the ordering field conjugate to the order parameter. This phenomenology was successfully applied to the description of the properties of supercooled water and several water-like models, such as TIP4P/2005 and S2.

Author Response:

We thank the reviewer for this suggestion, which points to a general, unstated, but useful connection. The generic phenomenology of Anisimov and coworkers describes fluid polyamorphism through two alternative mechanisms: a "discrete" mechanism, driven by thermodynamic equilibrium between two structurally distinct interconvertible molecular states with the interconversion fraction as the natural order parameter, and a "continuous" mechanism, associated with two-scale nonideality in the Gibbs energy, which does not require the existence of discrete alternative structural entities. An observation made by Anisimov and coworkers is that these two mechanisms can produce nearly identical macroscopic phase diagrams and thermodynamic anomalies, and that discriminating between them from macroscopic thermodynamic observables alone is difficult.

Our results speak directly to this open question. The absence of intermediate or out-of-distribution local environments along the LDA–HDA transformation pathway (Figure 4A,C) constitutes molecule-resolved evidence that polyamorphism in water glasses proceeds through redistribution between two structurally well-defined populations rather than through continuous structural evolution, which is consistent with the discrete mechanism. This is precisely the discrimination that macroscopic thermodynamics cannot provide but that a molecule-resolved structural classifier can. We have added discussion of this connection in both the Results and Implications sections of the revised manuscript.

> ***Page 6:***
> *The population-redistribution picture also connects to the phenomenology of fluid polyamorphism developed by Anisimov and coworkers [43], which distinguishes between a "discrete" mechanism, driven by interconversion between two structurally distinct molecular states, and a "continuous" mechanism arising from nonideality in the Gibbs energy, both of which can produce identical macroscopic signatures. The molecule-resolved probabilities $P_{\mathrm{LDA},i}$ and $P_{\mathrm{HDA},i}$ computed here are analogous microscopic realizations in the glass state, of the interconversion fractions of the liquid state, central to that framework. The absence of intermediate environments along the LDA-HDA transformation pathway indicates that the discrete mechanism operates in water's amorphous ices. Our results are consistent with theoretical descriptions of water based on two-state models [44, 45]*

and in the ***Implications*** *section:*

> ***Page 7-8:***
> *The locality of the LDA–HDA distinction, encoded within the first coordination shell, challenges the prevailing view that second-shell correlations are necessary to resolve amorphous water environments [17, 20]. In vitrified glasses, density fluctuations are arrested in distinct configurational basins, and differences in interstitial occupancy become sufficiently pronounced that first-shell descriptors alone suffice, suggesting that the requisite length scale for structural classification is state-dependent. This state-dependence suggests that extending the framework to equilibrium liquid environments where broader thermal fluctuations are expected to increase configuration overlap may require a larger or different descriptor set. Classification of LDL and HDL environments as the liquid–liquid critical point is approached represents a compelling direction for future work. The absence of intermediate structural populations along transformation pathways, established here with a classifier capable of explicitly detecting OOD configurations, constrains the energy landscape picture of glass–glass interconversion [42] and provides molecular-scale evidence for first-order character even where macroscopic observables vary continuously. Additionally,*

the molecule-resolved classification demonstrated here offers a general microscopic strategy for distinguishing the discrete and continuous mechanisms of polyamorphism [43].

Reviewer Comment:

In a relatively recent study (Singh et al., "Two-State Thermodynamics and the Possibility of a Liquid-Liquid Phase Transition in Supercooled TIP4P/2005," *J. Chem. Phys.* **144**, 144504, 2016), the authors compared the phenomenological order parameter (fraction of interconversion) with hypothesized microscopic features based on two different criteria: distance to the fifth nearest neighbor (d_5) and local structure index (LSI). The parameter d_5 contains information about the local structure up to the first coordination shell (3.5 Å) only. Comparison between the prediction based on d_5 and the phenomenological EOS revealed near perfect agreement. Structural information beyond the first shell was presumably obtained through LSI. Interestingly, the application of LSI did not help in comparison with the phenomenological EOS. In my opinion, this resonates with the major result of the paper under review.

Author Response:

We thank the reviewer for highlighting this parallel. Singh et al. demonstrated that d_5 *reproduces the phenomenological interconversion fraction of two-state thermodynamics for TIP4P/2005 liquid water, while LSI, performs worse. We agree that this resonates with our finding that LDA and HDA are resolvable within the first coordination shell. However, this picture requires careful reconciliation with other results in the literature. Studies of supercooled liquid water near the liquid-liquid critical point, including those of Martelli et al. and Donkor et al., find that second-shell information is necessary to distinguish low- and high-density liquid environments. We believe these findings are consistent when viewed through the lens of state-dependence. We suspect first-shell information mostly suffices in the kinetically arrested glass, where structural distributions are narrow and well-separated, and captures bulk thermodynamic trends in the liquid away from the critical point (as in Singh et al.), but near the critical point, where fluctuations diverge and the structural distinction between the two liquids becomes increasingly subtle, longer-range information becomes necessary. This state-dependent picture is articulated in our manuscript, and we have expanded our discussion of state-dependence in the Implications section to make this picture more explicit. The relevant edit is shown in the response to the last comment.*

3 Reviewer 3

Reviewer Comment:

Gallagher and co-workers present an analysis of simulated data of amorphous ices. In their work, local structural information is encoded as a structural descriptor typical for machine learning. Based on classification with a drastically reduced version of the descriptor, the authors report that the only two amorphous local structural classes, low-density (LDA) and high-density (HDA), can be found during the pressure-induced phase transition in between them. The authors conclude the simulated trajectories show a first order phase transition with hysteresis, and emphasize the role of an interstitial water molecule in the first solvation shell.

I have no doubt that a full ACSF, or some of the numerous other descriptors, would able to do such a classification after a suitable model selection procedure. This work concludes that it suffices to consider a small subset of interpretable features thereof. The authors base their analysis on descriptor features, selected without human bias, that can still be converted back to human-interpretable information. This aspect adds notable general value to the work. In my opinion, the authors correctly identify that the target of the investigation dictates which descriptor performs best, and that the obtainable information is also dictated by this choice.

The work is timely considering the ongoing discussion about amorphous phases of ice. I find the work methodologically interesting as well. However, some of the following points necessitate a major revision, before I can personally recommend publication.

Author Response:

We thank Reviewer 3 for the careful reading and the substantive methodological comments. We address each point in turn below and believe that the revisions, together with the clarifications provided here, fully resolve the raised concerns.

3.1 Major Comments

Reviewer Comment:

The results of the work rely on classifying OOD correctly. For training, the authors use data from the extrema of the pressure range where no ambiguity exists for LDA/HDA. However, the training data does not contain OOD structures, the hidden third class of the prediction output. While an ACSF descriptor of a high number of features would probably encode all relevant structural information, the authors exclude descriptor features based on data of these two phases (HDA/LDA) alone. This raises a question if the dropped features would trigger OOD if included in the analysis procedure.

Author Response:

We thank the reviewer for this perceptive comment, which identifies an important nuance in our approach. We wish to first clarify what OOD detection means within our framework. Rather than a trained three-class classifier, OOD detection emerges automatically from the class-conditional probability densities estimated from LDA and HDA training data (i.e., data from which there is no ambiguity exists for LDA/HDA). In the original formulation, a configuration is flagged as OOD if its joint likelihood falls below a percentile of 0.1 for training likelihoods of both classes simultaneously. Critically, this means that OOD detection is relative to the structural axes selected as most

informative for distinguishing LDA from HDA. Thus, as the reviewer pointed out, a structure that is anomalous along a descriptor axis that was excluded during feature selection because it carried low mutual information with the LDA/HDA class label may not reliably be flagged as OOD. This is a possible limitation of the approach. Excluded descriptors that are correlated with retained ones are truly redundant and pose no concern; however, descriptors that are simply uninformative for LDA/HDA discrimination could in principle be informative for detecting other novel structures.

Based on the reviewer comment, we have modified our approach to outlier detection in improve its sensitivity. This is done by operating directly on the per-descriptor kernel density estimates, $p_{a,y}(x)$. Rather than relying on class conditional probabilities, we label an environment an outlier if it falls below a threshold (i.e., 5×10^{-4}) for the kernel density estimates of both HDA and LDA along any of the considered structural descriptors. The value 5×10^{-4} is somewhat arbitrary, but results are robust within a reasonable range as noted in the supporting information.

We also updated the algorithm to broaden the set of descriptors over which OOD detection is evaluated. Classification of LDA and HDA continues to use the five most informative, weakly correlated descriptors identified by the mutual-information/correlation-filter procedure, which preserves the interpretability of the classifier. For detecting outliers, however, we now evaluate the criterion above over every descriptor retained by the correlation filter. This substantially increases the sensitivity of OOD detection without compromising the interpretability of the classification step itself. We find that this improves the OOD detection capability of our models, as described in Section S4 of the Supporting Information.

To this end, we have edited the description of our method in the SI accordingly:

> ***Section S1B, page 5:*** *To ensure that selected descriptors contribute distinct structural information, we use a greedy high-correlation filter. Proceeding in order of decreasing MI, each descriptor is retained only if its Pearson correlation (r) with all previously retained descriptors satisfies $|r| < 0.8$. The outcome is a set of informative and roughly uncorrelated descriptors, which we denote $\mathcal{D} = \{1, \ldots, M\}$. Because the procedure operates on arbitrary descriptor inputs, it enables data-driven identification of discriminative descriptors without requiring prior assumptions about which structural motifs are important. We retain $\mathcal{D}$ in its entirety for out-of-distribution detection (see Probabilistic Model, below), since each additional weakly correlated descriptor offers an independent opportunity to identify an anomalous environment and thus maximizes detection sensitivity. For classification, however, the number of descriptors retained from $\mathcal{D}$ is a user-specified parameter; here we select the $m = 5$ most informative descriptors, denoted $\mathcal{D}_{cls} = \{1, \ldots, m\} \subset \mathcal{D}$, balancing classification accuracy with physical interpretability. The full set of ACSF and BOO descriptors considered as candidates spans all functional forms defined in Section SI 1A, and the specific descriptors selected for LDA/HDA classification are shown in Figure 2E of the main text.*

> ***Section S1C, page 5:*** *A further advantage of this probabilistic formulation is explicit out-of-distribution (OOD) detection. To maximize sensitivity, OOD detection is evaluated over the complete descriptor set $\mathcal{D} = \{1, \ldots, M\} \supseteq \mathcal{D}_{cls}$ (see Automatic Feature Selection, above) rather than the m descriptors used for classification alone. For each descriptor $d \in \mathcal{D}$ and class y, we construct $p_{d,y}(x)$ following the same KDE procedure described above, with bandwidth σ_d selected independently via the same five-fold cross-validated grid search. We define an environment as an outlier if $p_{d,y}(x) < P_{cut}$ for all classes y, for any descriptor $d \in \mathcal{D}$. This criterion ensures that the vast majority of in-distribution*

> *configurations are retained while enabling detection of novel or anomalous environments, a capability essential for analyzing structural transformations. In this work, we choose $P_{cut} = 5 \times 10^{-4}$. We find that our results are robust up to $P_{cut} = 1 \times 10^{3}$, beyond which outlier configurations are observed during compression/decompression trajectories. This threshold can be adjusted to make the classifier more or less conservative, depending on the application requirements.*

We have also updated the main text to clarify the method change and have adjusted all results dependent on outlier detection. We now state that:

> ***Methods, page 9:*** *The most discriminative descriptors are identified through a two-stage selection procedure. First, each descriptor is ranked by its mutual information (MI) with the class label [53], retaining those with MI values at least 10% of the maximum observed value. Second, a greedy correlation filter removes redundant descriptors. Proceeding in order of decreasing MI, a descriptor is retained only if its Pearson correlation with all previously retained descriptors satisfies $|r| < 0.8$. This yields a compact set of informative, approximately uncorrelated descriptors without requiring prior assumptions about which structural motifs are important. For classification, we use the five most informative of these descriptors (Fig. 2E); for OOD detection (below), we instead use the complete correlation-filtered descriptor set, as additional weakly correlated descriptors provide independent axes along which an anomalous environment may be identified and thereby maximize outlier detection sensitivity.*

Figures 4A and 4C have been updated with no qualitative changes. We note that including all structural descriptors for OOD identification does not lead to any significant increases in the population of outlier configurations during HDA/LDA transitions, reaffirming the first-order nature of the transition.

Reviewer Comment:

The same cross-validation is used for training and for evaluation of performance. The orthodox way to study performance is to split the data to a separate training and test part to avoid leakage of information. If any choice or step is based on CV score for training, then information leaks from test to training.

Author Response:

We agree that test set evaluation must be handled with care to avoid data leakage and an inflated assessment of model generalization. In our case, we do not use CV scores for any "choice or step" but rather use CV as a means of estimating the error of our models on unseen data using several train/test splits.

The original manuscript stated that kernel bandwidths are estimated using five-fold cross-validation, and this may have been interpreted as using the same aforementioned cross-validation procedure to obtain model hyperparameters and generalization accuracy. This is not the case; we use a separate cross-validation procedure on the training data (i.e, a nested cross-validation loop) to estimate kernel bandwidths. A separate (outer) cross-validation loop is used to assess model accuracy; so, we have a nested structure. We have edited the manuscript to clarify.

> ***Methods, page 9:*** *Classification proceeds by modeling the class-conditional distribution of each selected descriptor independently using Gaussian kernel density estimation (KDE), with bandwidths optimized by five-fold cross-validation on the provided training data.*

Reviewer Comment:

Since the work relies on LDA/HDA/OOD classification, a notable fraction of the test set should consist of OOD structures of relevant local densities. In particular this calls for high pressure ice phases and the liquid structures at high pressures, that are reasonably close in the local configuration space. As a consequence of the present choice, the reported accuracy 99.3% is skewed to the optimistic side due to selection bias toward extreme LDA/HDA cases. More critically, evidence for performance is largely missing for the OOD structures, apart from a test for 1000 structures of hexagonal ice. While the spirit of this test is to be appreciated, one has to remember that the structural descriptor vector has been heavily truncated based on data for only LDA and HDA.

Author Response:

The reviewer is correct in that our demonstration of effective outlier detection is limited to only hexagonal ice structures. We have addressed this by (i) expanding our OOD test set to 14 additional crystalline ice polymorphs spanning a wide range of pressure-stabilized structures and (ii) directly comparing OOD detection using only the five descriptors selected for LDA/HDA classification against the complete set of weakly correlated descriptors retained by the correlation filter described in Methods. This new analysis is reported in a new SI section (SI Sec. 4), reproduced in full below, and is referenced in the main text.

Table 1 below shows five phases (Ih, Ic, VII, VIII, and XI) are already detected with near-perfect accuracy using only the five LDA/HDA-discriminating descriptors, indicating that these phases are structurally distinct from LDA/HDA even along this minimal set. Six phases (III, V, IX, XII, XIII, and XIV) improve dramatically when the full descriptor set is used, from 0–31% detection with the five-feature model to 76–100% detection with the complete set. This is direct evidence that a descriptor vector selected specifically to discriminate LDA from HDA is, as the reviewer anticipated, not ideally suited to detecting structural deviations along unrelated directions in configuration space. The remaining four phases (II, IV, VI, and XV) improve only partially. This indicates that the local environments of these particular high-pressure polymorphs are not well separated from LDA/HDA along any of the set descriptors conditioned as most informative for LDA/HDA classification, considered here. Improving sensitivity to these residual cases, for example, through additional descriptor families, is a natural direction for future work. We thank the reviewer for identifying this shortcoming, which has improved the reliability of our method.

The reviewer additionally highlights high-pressure liquid environments as a natural complement to the high-pressure crystalline phases considered above, given their proximity to LDA/HDA in local configuration space. Rigorously evaluating OOD detection for liquid water would require additional reference trajectories spanning a range of pressures, and is closely related to the question of classifying LDL- and HDL-like environments as the liquid–liquid critical point is approached, which we identify as a compelling direction for future work in the Implications section of the main text.

> ***SI Section 4: Out-of-distribution Prediction for Alternative Phases of Ice***
>
> *Our outlier detection method identifies local atomic environments that differ from the LDA/HDA training distribution along any structural dimension. To evaluate how the choice of descriptor set affects OOD detection, we compare two approaches applied to an expanded dataset of ice polymorphs obtained from the datasets used to train the DP_SCAN and DP_MBpol models [37, 39].*
>
> *The first approach uses only the five descriptors in $\mathcal{D}_{cls}$ selected for LDA/HDA classification (see Automatic Feature Selection). As discussed in the main text, this yields a sparse, interpretable model whose features directly reflect what distinguishes the two amor-*

phous states from one another. The second approach uses the complete correlation-filtered descriptor set $\mathcal{D}$ (descriptors retained after removing pairwise-correlated candidates with Pearson $|r| > 0.8$). This set is not equivalent to using every candidate descriptor simultaneously: including strongly correlated descriptors in a naïve Bayes product model would double-count shared structural information and degrade, rather than improve, OOD sensitivity.

Table 1: Accuracy of classifiers that use different numbers of features for OOD classification. Models are used to classify structures of different ice phases generated by the DP_MBpol and DP_SCAN water models. Models can either use five features relevant to LDA/HDA classification ($n = 5$) or use all uncorrelated provided structural descriptors ($n = 120$).

Phase	DP_MBpol		DP_SCAN	
	$n = 5$	$n = 120$	$n = 5$	$n = 120$
Ice Ih	1.000	1.000	1.000	1.000
Ice Ic	1.000	1.000	0.998	0.998
Ice II	0.000	0.122	0.003	0.378
Ice III	0.009	0.933	0.001	1.000
Ice IV	0.012	0.033	0.048	0.071
Ice V	0.003	0.872	0.064	0.998
Ice VI	0.024	0.134	0.087	0.250
Ice VII	1.000	1.000	1.000	1.000
Ice VIII	1.000	1.000	1.000	1.000
Ice IX	0.005	0.993	0.000	1.000
Ice XI	0.993	0.998	1.000	1.000
Ice XII	0.062	0.991	0.300	1.000
Ice XIII	0.006	0.763	0.052	0.980
Ice XIV	0.031	0.987	0.312	1.000
Ice XV	0.061	0.214	0.264	0.470

Table 1 shows OOD detection rates for both approaches across 14 ice polymorphs. Using the full uncorrelated descriptor set outperforms the five-feature model in most cases, with several phases moving from near-zero to near-complete detection. This indicates that the combined ACSF and BOO descriptor set contains structural information sufficient to distinguish the majority of ice polymorphs from amorphous ice. A subset of high-pressure phases (e.g., Ice II, IV, VI, and XV) remain only partially distinguishable from LDA/HDA even when using the full descriptor set.

This residual ambiguity is a consequence of the descriptor selection procedure used here, rather than the local environment representation itself. At the level of individual atomic neighborhoods, some high-pressure ice phases share geometric features with LDA/HDA that are not fully resolved by the descriptors identified that optimally distinguish LDA and HDA. This does not indicate that these crystalline phases are physically present within computational LDA or HDA. Independent molecular dynamics studies of LDA and HDA have found no evidence of ice-like domains in either amorphous phase [40]. Taken together, these results suggest that the observed classifier ambiguity for certain high-pressure ices arises from local geometric similarity between amorphous and crystalline packing motifs along the LDA/HDA classification axis, rather than from partially crystallized regions embedded within the amorphous structures. Distinguishing such phases would be possible if a classifier were directly trained to resolve the amorphous states from the crystalline

ones.

Regarding the comment on classification accuracy, we agree that the reported accuracy of 99.3%, a binary classification accuracy for HDA and LDA, is optimistic if one's end goal is to classify environments encountered in simulations that may differ from HDA and LDA structures. In the original manuscript, this was acknowledged with a note about limitations of classification accuracy, and we have also clarified that this is only for binary classification:

> ***Page 3:*** *All approaches achieve high classification accuracy, confirming that LDA and HDA environments are microscopically distinguishable. Our method achieves 99.3% binary classification accuracy, matching or surpassing all benchmarks and outperforming classifiers trained on BOO parameters or ACSFs alone, indicating that combining complementary descriptor types improves classification. However, high classification accuracy alone does not guarantee reliability when applied to transformation trajectories, where configurations belonging to neither LDA nor HDA may arise.*

Reviewer Comment:

Given the spatial limitations of the selected features, how would the authors even detect a change further from the origin? In other words, the question setting limits the possible conclusions to the first solvation shell, without a test showing that such a cutoff would be suitable for the studied question. This point could be addressed by slight re-wording in the manuscript.

Author Response:

We interpret this comment as noting that the fixed descriptor cutoff radius ($R_c \approx 5$ Å, corresponding to 16 nearest neighbors) bounds the conclusions that can be drawn about length scales. Since no structural information beyond this cutoff is explictly presented to the classifier, one cannot rule out that longer-range information would further improve classification. We agree, and have reworded the relevant claims in the manuscript accordingly. The conclusion should be understood as: sufficient information for accurate classification is available within the environment size considered, not that no structural differences exist at longer range.

We also note that the full descriptor set includes locally-averaged BOO parameters ($\bar{q}$, $\bar{w}$), which incorporate structural information from beyond the first coordination shell by averaging orientational order over a central molecule and its neighbors, providing implicit sensitivity to longer-range structural correlations. Despite this, the environment-size analysis of Figure 3A, which was performed using strictly local descriptors explicitly excluding these averaged quantities, still achieves accurate classification with a clear plateau before the maximum environment size is reached. The fact that accuracy saturates rather than continuing to improve with environment size demonstrates that there is no motivation to extend the cutoff further. A key practical advantage of the present framework is that this length-scale dependence is trivially and efficiently evaluated, and we recommend performing this scan as a standard first step for any new application to identify the optimal environment size for the problem at hand, as the saturation behavior may be system-specific.

Finally, we wish to clarify the nature of the scientific question being addressed. LDA and HDA are trivially distinguishable globally by bulk density and scattering data. The non-trivial question we address is whether individual molecular environments can be probabilistically assigned to the LDA or HDA structural manifold, which stresses locality. OOD detection follows naturally. The spatial locality of the descriptors therefore reflects the nature of the question being asked rather than a limitation of the approach. We have revised the manuscript as follows:

Page 5: *These conclusions are necessarily bounded by the maximum environment size considered and do not exclude the possibility that structural differences between LDA and HDA persist at longer range. The length-scale scan of Figure 3A is computationally inexpensive and constitutes a useful diagnostic for any new application of this framework, revealing the environment size beyond which additional structural information ceases to improve discrimination; this saturation behavior may be system-specific. More broadly, the question addressed here is inherently local. LDA and HDA are readily distinguishable by their bulk properties, and the non-trivial result is that this distinction is already encoded at the level of individual molecular environments.*

3.2 Minor Comments

Minor Comment:

The caption for Figure 4F does not explain the plot sufficiently. While the pair of curves high in G^1_{H} are clear, the description of the curves with lower G^1_{H} feature values is absent. From the text on page 7 ("when G^1_{H} is replaced by q_4") I deduce that these curves represent a projection on the q_3–q_4 plane.

Response:

The lower set of curves in Figure 4F represents the compression and decompression trajectories projected onto the q_3–q_4 plane (i.e., with G^1_{H} replaced by q_4), plotted alongside the q_3–G^1_{H} trajectories above. We have clarified panel F of the caption to explicitly describe both sets of curves shown in the 3D plot and reference the 2D projections in the SI.

> ***Figure 4 Caption:***
> ***(F)*** *Mean local-environment descriptors (q_3, q_4, G^1_{H}) along compression (blue) and decompression (red) pathways for separate isothermal MD simulations at temperatures from 60 to 140 K. The upper set of curves shows trajectories in the full q_3–q_4–G^1_{H} space; the lower set shows the same trajectories projected onto the q_3–q_4 plane. Stars mark transition points; darker shades correspond to lower temperatures. See SI Figures S2 and S3 for 2D projections.*

Minor Comment:

Is the "temperature" cited for curves of Figure 4F evaluated for a local environment, or are these results from separate MD runs?

Response:

The temperatures in Figure 4F correspond to separate isothermal MD compression and decompression simulations performed at each temperature, i.e., they are global simulation temperatures, not quantities evaluated for individual local environments. This is noted in the revised caption above.

Minor Comment:

On page 6, right column: "structurally distinct regions of configuration space" might need "local", as the analysis is carried out for a maximum of 17 water molecules.

Response:

We thank the reviewer for this careful reading. We have revised the phrase to "structurally distinct regions of local configuration space" to clarify that the statement refers to the space of local molecular environments.

Minor Comment:

SI section A, phrasing: A molecule cannot be classified as LDA/HDA. A sufficiently large local environment may be.

Response:

Agreed. We have amended the text to refer to a molecule's local environment and to refer to the environment as LDA- or HDA-like at certain locations.

Minor Comment:

SI page 5: a broken reference.

Response:

The broken reference has been corrected in the revised SI.

Minor Comment:

The MD section lacks details such as the timestep, the system size, the ensemble, and preparation of the initial state of the system.

Response:

The MD simulation details (timestep, system size, ensemble, and initial state preparation) are fully reported in prior publication by some of us (Szukalo, Giovambattista, and Debenedetti, Proc. Natl. Acad. Sci. U.S.A. ***122****, e2509609122, 2025), to which we refer in the Methods section. We have added a summary of these details directly to the SI.*

> **SI Section 1D, Page 6:** All configurations were obtained from the Deep Potential (DP) MD trajectories reported in Ref. 24. Two machine-learned potentials were employed, providing complementary representations of water's potential energy surface. The first, DP_SCAN, was trained on electronic-structure data generated with the SCAN density functional [38, 39], a meta-GGA functional known to reproduce many of water's structural and thermodynamic properties. The second, DP_MBpol, was trained on the many-body polarizable (MBpol) potential [37, 46–48], which is parameterized against coupled-cluster reference data and is among the most accurate models of water available. All simulations were performed using LAMMPS (2 Aug 2023) [49] interfaced with DeePMD-kit (v3.0.0) [50, 51], with a timestep of 0.5 fs. Simulations employed a system of 512 water molecules in a cubic box with periodic boundary conditions and the temperature and pressure were controlled via a Nosé–Hoover thermostat and barostat, respectively.
>
> LDA configurations were generated by isobaric quenching from equilibrated liquid water configurations at constant cooling rates of $q_c = 1$ K/ns and $q_c = 10$ K/ns, at 1 bar. Isothermal compression and decompression simulations were conducted starting from LDA configurations at constant rates of $q_p = 300$ bar/ns or $q_p = 3000$ bar/ns. For each pressure and cooling rate, at least three independent simulations were performed to assess statistical variance.
